# Thermodynamic Basis for the Emergence of Genomes during Prebiotic Evolution

**DOI:** 10.1371/journal.pcbi.1002534

**Published:** 2012-05-31

**Authors:** Hyung-June Woo, Ravi Vijaya Satya, Jaques Reifman

**Affiliations:** DoD Biotechnology High Performance Computing Software Applications Institute, Telemedicine and Advanced Technology Research Center, United States Army Medical Research and Materiel Command, Fort Detrick, Maryland, United States of America; University of Washington, United States of America

## Abstract

The RNA world hypothesis views modern organisms as descendants of RNA molecules. The earliest RNA molecules must have been random sequences, from which the first genomes that coded for polymerase ribozymes emerged. The quasispecies theory by Eigen predicts the existence of an error threshold limiting genomic stability during such transitions, but does not address the spontaneity of changes. Following a recent theoretical approach, we applied the quasispecies theory combined with kinetic/thermodynamic descriptions of RNA replication to analyze the collective behavior of RNA replicators based on known experimental kinetics data. We find that, with increasing fidelity (relative rate of base-extension for Watson-Crick versus mismatched base pairs), replications without enzymes, with ribozymes, and with protein-based polymerases are above, near, and below a critical point, respectively. The prebiotic evolution therefore must have crossed this critical region. Over large regions of the phase diagram, fitness increases with increasing fidelity, biasing random drifts in sequence space toward ‘crystallization.’ This region encloses the experimental nonenzymatic fidelity value, favoring evolutions toward polymerase sequences with ever higher fidelity, despite error rates above the error catastrophe threshold. Our work shows that experimentally characterized kinetics and thermodynamics of RNA replication allow us to determine the physicochemical conditions required for the spontaneous crystallization of biological information. Our findings also suggest that among many potential oligomers capable of templated replication, RNAs may have evolved to form prebiotic genomes due to the value of their nonenzymatic fidelity.

## Author Summary

A leading hypothesis for the origin of life describes a prebiotic world where RNA molecules started carrying genetic information for catalyzing their own replication. This origin of biological information is akin to the crystallization of ice from water, where ‘order’ emerges from ‘disorder.’ What does the science of such phase transformations tell us about the emergence of genomes? In this paper, we show that such thermodynamic considerations of RNA synthesis, when combined with kinetics and population dynamics, lead to the conclusion that the ‘crystallization’ of genomes from its basic elements would have been spontaneous for RNAs, but not necessarily for other potential building blocks of genomes in the prebiotic soup.

## Introduction

All biological organisms are evolutionarily related. The salient characteristics of life (reproduction and selection) must have therefore emerged either gradually or abruptly from inanimate chemical processes some time in the early history of the Earth. Our ever-increasing knowledge on the biochemical and genetic basis of modern life forms should guide the quest to understand this transition, in addition to the chemistry of potential building blocks [1], [2] and geochemical considerations [3], [4]. The lack of fossil evidence forces us to rely on model building, which can often be tested experimentally in the laboratory [5]. One of the simplest and most promising is the RNA world hypothesis [1], [6], [7], which proposes RNA molecules as precursors to modern life forms consisting of DNAs as carriers of genomes and proteins as molecular machines. Continued progress in experimental studies has yielded a diverse range of evidences supporting this hypothesis. In particular, plausible synthetic routes to nucleotides [2] and oligomers [8] have been demonstrated. RNA ribozymes capable of catalyzing RNA replications have been designed and synthesized via *in vitro* selection [9],[10]. Extensive studies of RNA folding landscapes further demonstrate the capability of RNAs to function both as carriers of genotypes and phenotypes [11], [12].

Conceptual difficulties to this scenario include the need for the existence of sufficiently concentrated and pure building blocks (chirally selected nucleotides for RNAs) and the necessity to explain subsequent evolutions of multi-chemical autocatalytic systems [13]: the incorporations of proteins and nonreplicative metabolic networks. In this context, Nowak and Ohtsuki recently considered a model describing a pre-evolutionary stage with nonreplicative chemical selection [14]. The undeniable strength of the RNA world hypothesis, nevertheless, is that it has the potential to provide an empirically well-tested pathway for the transition from chemistry to biology, irrespective of its factual historical relevance. The relative simplicity of the model should also allow quantitative descriptions that can complement empirical approaches.

Our focus in this paper, in particular, is the transition from the first RNA molecules formed, which must have been pools of near-random RNA sequences, to the first genomes coding for RNA ribozymes. Crucial in understanding such an emergence of the first RNA genomes is the error threshold predicted by the quasispecies theory [15]–[17]. At this threshold, the structure of a population of RNA sequences shifts from being dominated by a stable genome (‘master sequence’) to becoming random pools, or vice versa. This transition can also be described and understood in the context of more general population dynamics models [18], [19], for which many exact results have now been obtained based on statistical physics approaches [17], [20]–[24]. The error catastrophe transition is in the forward direction, and has thus been likened to ‘melting’ by Eigen [15]. The transition has recently been observed in behaviors of modern RNA viruses exposed to mutagens [25], [26]: a moderate artificial increase in mutation rates of viruses can lead to a complete extinction of virus populations. The error threshold is roughly proportional to the inverse of genome length, which also raised the question of how genomes long enough to encode error correction could have evolved under high error rates (Eigen's paradox) [15], [27], [28]. Notably, Saakian et al. [29] have recently applied analytical treatments of quasispecies theory to consider this question. Higher organisms keep error rates down to levels that are orders of magnitude lower than achievable by polymerases only, using sophisticated error correction mechanisms including mismatch repair complexes. Tannenbaum et al. [30], [31] have studied the quasispecies models of organisms posessing mismatch repair genes, finding transitions analogous to the classic error catastrophe transition in repair-deficient mutator frequencies.

The prebiotic evolution in the RNA world is in the opposite direction of the error catastrophe transition, and may thus be referred to as ‘crystallization.’ In an equilibrium fluid, whether one observes melting or crystallization is determined by the changes in temperature and pressure. Can we find analogous conditions for the emergence of the first genomes? Addressing this question requires connections to thermodynamics of RNA synthesis. Recent developments in the theory of nucleotide strand replication [32]–[34] provide a promising new direction to bridge the gap between the basic chemical thermodynamics of RNA synthesis and molecular evolution. The mean error rate of replication increases as the reaction condition approaches equilibrium, contributing to entropy production [32]. With a combination of this single-molecule thermodynamics and quasispecies theory, a surprisingly complete analogy to equilibrium fluids was proposed [34], where volume, pressure, and temperature are replaced by replication velocity, thermodynamic force, and inverse fidelity, respectively, with counterparts of condensation, sublimation, critical point, and triple point. Based on the analysis of a model replication kinetics equivalent to the Jukes-Cantor model of DNA evolution [35], it was suggested that the prebiotic evolution of RNA strands may have been biased by a thermodynamic driving force toward increasingly higher fidelity of polymerase ribozymes below a certain threshold [34].

To what extent these theoretical predictions are applicable to the actual prebiotic evolution that occurred in the past must ultimately be judged based on quantitative empirical data from existing and new experiments. Here, we extend our previous work [34] and assess the applicability of this thermodynamic theory of molecular evolution to prebiotic evolution, using experimental data for polymerization kinetics currently available in the literature. Our results based on these empirical data provide a strong support for the main conclusion of the theory, that there is a thermodynamic driving force biasing random sequence evolutions in the absence of genomes toward higher fidelity in a certain regime of parameter spaces. With considerations of the time-dependent evolutionary behavior of RNA populations, we furthermore show that it is possible to estimate the time scales that would have been required for a random sequence pool to crystallize a newly discovered master sequence under a given thermodynamic condition. These results also shed new light on Eigen's paradox. Most importantly, our approach enlarges the scope of both the quasispecies theory-based discussions of the stability of genomes and biochemical approaches to RNA replication by introducing the concept of thermodynamic driving forces and constraints in molecular evolution.

## Results/Discussion

### RNA replication kinetics and thermodynamics

**Figure 1:**
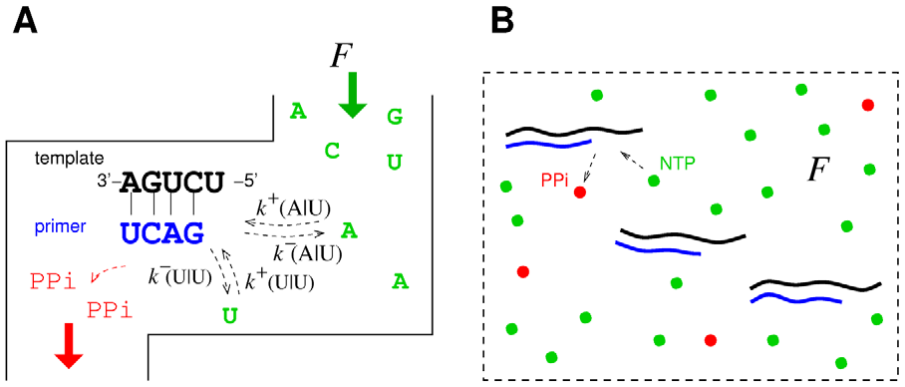
Kinetics and thermodynamics of RNA replication. A: Single-molecule kinetics. B: Population dynamics.

The thermodynamic theory of molecular evolution [34] combines the kinetics and thermodynamics of RNA replication on a single-molecule level with population-level features. We first consider the molecular level description of RNA synthesis (or elongation): an elementary step of insertion by addition of a nucleotide (Figure 1A) consumes a nucleoside triphosphate (NTP) and produces a pyrophosphate (PPi). Its driving force 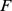 is given by
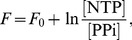
(1)where 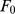 is defined such that 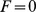 at equilibrium (see Methods). We may estimate the equilibrium constant from 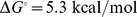 of DNA phosphodiester bond formation and 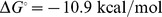 of 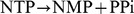 (NMP: nucleoside monophosphate), yielding 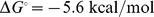 [36], [37]. This value likely overestimates the magnitude of 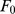 because it ignores the unfavorable entropy change of binding a free NTP monomer, leading to 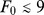.

One may seek the origin of the observed high-fidelity of polymerization reactions [38] in the relative thermal instability of incorrectly formed Watson-Crick base pairs. However, the stability differences between correctly and incorrectly inserted nucleotide pairs are small: an experimental estimate based on melting temperature measurements for the difference in free energy between incorrect and correct pairs yielded 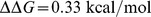 [39], which we adopted in this work. This value is the average of the relative stabilities of nucleotides G, C, and T (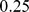, 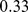, and , respectively [39]) with respect to A in a DNA 9-mer duplex terminus against the template base T. The precise value depends on the identity of the base pairs at the terminus and at the neighboring position immediately upstream: for DNAs, duplex stabilities including effects of mismatches can be reliably estimated based on nearest neighbor interactions [40]. Longer-ranged interactions presumably play more important roles for RNAs, which form secondary structures and higher-order folds [41], affecting 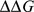 values. Frier et al. [42] provided values of free energy contributions to the duplex stability from all 16 possible terminal RNA base pairs and mismatches next to 4 distinct base pairs upstream (Table 4 in Ref. [42]). From these data, we calculated 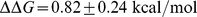, comparable to 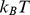.

In quantitative descriptions encompassing both the high kinetic selectivity and this marginal stability difference, it is important to fully take into account the reversibility of the reactions [32]. We adopt the simplest description of the kinetics of polymerization, specified by 16 forward and reverse rates, 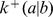 and 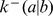, respectively, each corresponding to the insertion and its reverse of a nucleotide (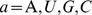) against a template base (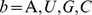; Figure 1A). In reality, these rates do depend on the identity of base pairs immediately upstream [40], [41], which may lead to stalling after incorrect incorporations [28]. More importantly, however, these rates also depend on 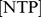 and 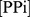. We estimated the forward rates from the available experimental data of primer extension under the far-from-equilibrium limiting condition [9], [28], [43]–[49]. The backward rates can then be related to the forward rates via equilibrium stability.

In general, the overall elongation reaction of a single nucleotide goes through a transition state, whose activation energy is differentially affected by the action of polymerases. If one ignores the reverse reaction under the condition of 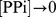, the Michaelis-Menten kinetics applies for the primer extension. In the limit of small 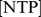, we then have 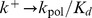, the latter representing the apparent second-order rate constant with the substrate dissociation constant 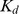 and the turnover rate of product formation 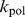 [50]. Measurements of polymerase-catalyzed reactions show the selectivity reflected in differences in 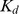 for correct and incorrect base pairs to be orders of magnitude larger than equilibrium stability differences [39]. Examples currently found in the literature are shown in Tables 1 and 2, including those for activated nonenzymatic polymerization (DNA replication without enzymes) determined recently by Chen et al. [28]. Table 1, in particular, shows the dramatic increase in the degree of relative stabilization of the transition states for correct base pairs in modern polymerases. The evolution of polymerases has entailed two aspects: the facilitation of the overall elongation rate and the amplification of the preferential attachment of correct versus incorrect nucleotides. As we show below, this latter aspect of selectivity evolution leads to a phase transition-like behavior, profoundly affecting population dynamics of evolving macromolecules.

**Table 1:** Reference base incorporation rates 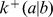 of NTPs (rows) against template bases (columns).

A. Nonenzymatic [28]				
	A	T	G	C
ATP	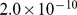	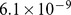	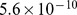	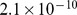
TTP	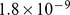	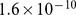	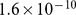	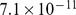
GTP	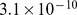	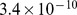	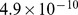	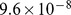
CTP	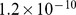	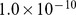	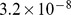	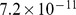

Rates are defined as the apparent second order rate constant 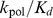 (or the limit of 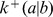 for small [NTP]) in units of 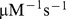.

1 For poliovirus 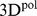, the mismatch rate has been reported for only one combination 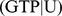. We assumed that the same ratio 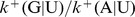 applies to all NTPs for each template base. The value for 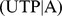 is a harmonic mean of two data (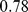 and 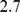).

**Table 2:** Reference base incorporation rates for DNA polymerases.

A. *Sulfolobus solfataricus* P2 DNAP IV (Dpo4) [44]				
	A	T	G	C
ATP	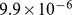	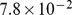	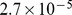	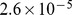
TTP	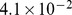	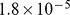	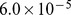	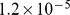
GTP	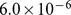	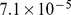	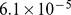	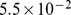
CTP	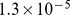	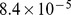	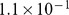	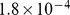

Rates are defined similarly in the same units as in Table 1.

1 For pol 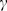, it was assumed that 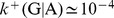.

**Figure 2:**
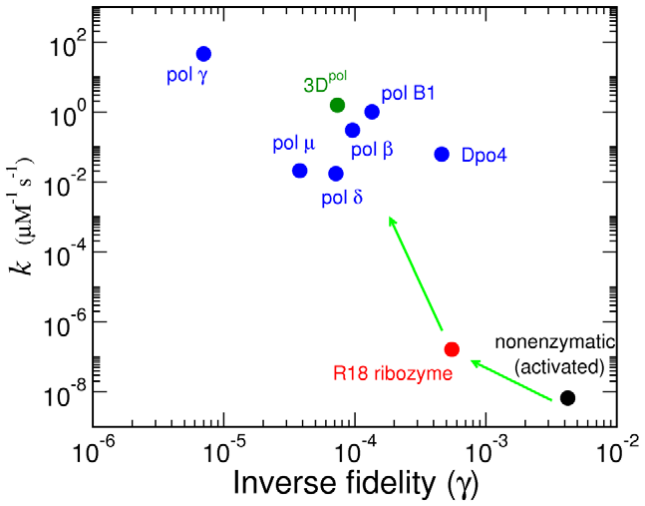
Variations of inverse fidelity 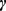 and mean base incorporation rate 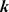 among polymerases. See Tables 1 and 2 for the references. Arrows show the likely direction of evolutionary changes.

To characterize this dual aspect of enzyme-catalyzed polymerization reactions, we adopt a ‘reduced’ description involving two key characteristics of forward rates: the mean base incorporation rate 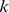 and the relative inverse fidelity 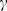 (the ratio of incorrect to correct insertion rates). Precise definitions of these quantities in terms of kinetic rates emerge from the mean field theory (see Methods):
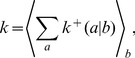
(2a)

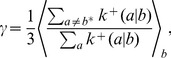
(2b)where 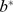 is the Watson-Crick complementary base of 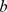 and the angled brackets denote a harmonic mean over distribution 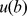 of template bases:
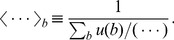
(3)
Figure 2 shows the distribution of these quantities among nine polymerase systems whose polymerization kinetics have been determined experimentally (Tables 1 and 2), in which we observe qualitative trends of the evolutionary changes reflected on the values of 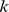 and 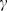: the 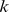 values of modern polymerases are 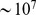 times larger than the activated nonenzymatic rate, while the nonenzymatic fidelity (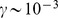) implies that the Watson-Crick structure in the absence of enzymes already supports a fairly high level of fidelity. The arrows illustrate the direction of evolutionary changes that must have occurred from the nonenzymatic to protein-based polymerases via the polymerase ribozymes in the RNA world.

The nonenzymatic data are for the templated oligomerization of activated nucleotide analogs, the nucleoside 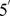-phosphorimidazolide, where PPi is replaced by the imidazole group [28]. Zielinski et al. have compared the kinetics of RNA versus DNA elongation of the activated system [51]. They concluded that RNA elongation is more efficient because its A-form helical structure positions the 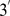-OH group towards the incoming monomer, whereas contributions of wobble-pairing appeared to facilitate mismatches. This study suggests that the nonenzymatic kinetic rates for RNAs may have higher 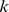 and 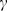 values than for DNAs. We nevertheless expect their order of magnitudes to be similar.

### Mean field theory

**Figure 3:**
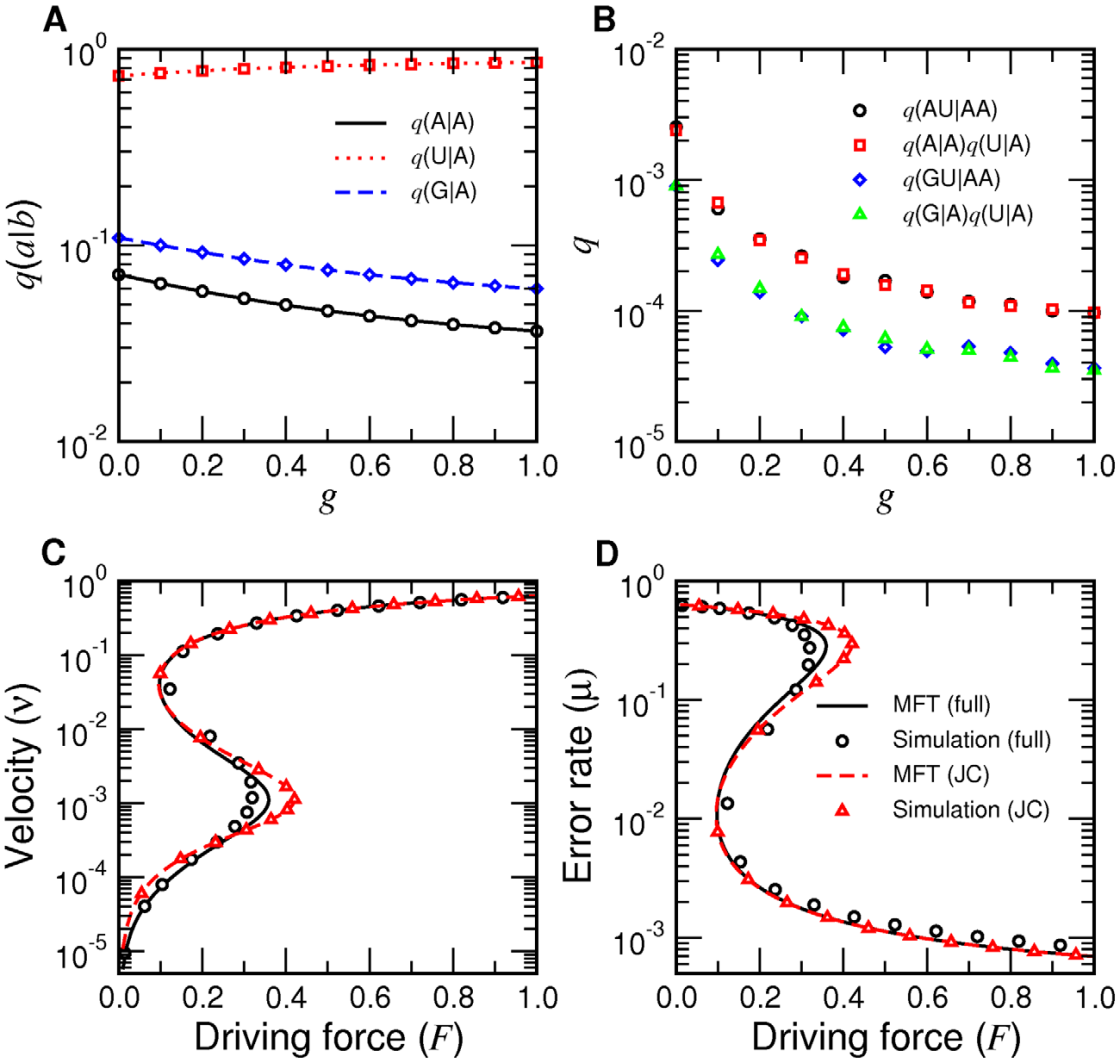
Numerical tests of mean field theory. A: Three components of 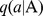 as functions of 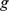 for a symmetric template model for which the mean field theory is exact. The rates were given by 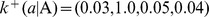, 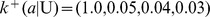, 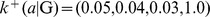, and 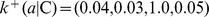 for 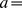
A, U, G, C. Lines are from Eq. (40). Symbols are from numerical simulations. B: Test of site-independence for the sequence distribution, Eq. (35), with pol 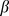 rates (Table 2F). All symbols were calculated from numerical simulations. C–D: Mean velocity (C) and error rate (D) for the pol 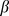 kinetics, both with full experimental kinetics (Table 2) and Jukes-Cantor version (JC) derived from the full kinetic set. Symbols are from simulations, which verify that for JC kinetics the mean field prediction is exact.

The kinetic rates and thermodynamic conditions (the value of 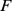) allow us to extract, using simulations in general (see Methods), the main stationary properties of RNA elongation: the mean elongation velocity 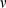 (the average number of nucleotide pairs added per unit time) and error rate 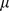 (the average fraction of mismatched nucleotide pairs). They differ from their respective microscopic counterparts, 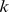 and 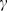, because of varying contributions of the reverse rates as functions of 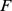. Importantly, exact analytic expressions for the stationary properties can be obtained if the kinetic rates have sufficient symmetry: the set of 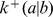 for all 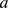 is independent of the identity of 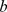 (‘symmetric template models;’ see Methods and Figure 3).

In Ref. [34], an important special case of symmetric template models
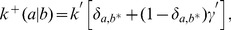
(4)equivalent to the Jukes-Cantor model of DNA evolution [35], was considered. The Jukes-Cantor model is a two-parameter model, while general symmetric template models have four parameters.

However, to quantitatively assess the applicability of the theory based on empirical data of RNA replication kinetics, it is necessary to allow all 16 values of 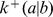 to be independent empirical parameters. Here, we used a version of the mean field theory that generalizes the analytic results with the following expressions for the elongation velocity 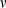 and error rate 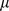 (see Methods):
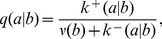
(5a)

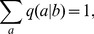
(5b)

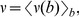
(5c)

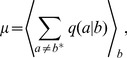
(5d)where 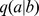 denotes the probability to find 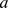 base-paired to 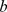, and Eq. (5b), the normalization condition for 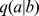, determines 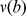, the mean velocity of nucleotide addition against template base 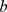. Equation (5a) is a generalization of the equilibrium Boltzmann distribution, to which it reduces to when 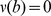, and is exact for symmetric template models (Figure 3). Because the complete reproduction of an RNA strand requires a pair of replications, we also considered the net error rate 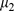 of two consecutive replications:
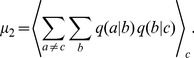
(6)


For connections to thermodynamics, one must calculate the entropy production (in units of 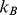) per monomer addition [32]:
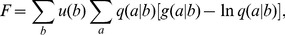
(7)where the first term in the square brackets represents the contribution of monomer consumptions to dissipation and the second term corresponds to the disorder creation by copying errors. The average in Eq. (7) is an arithmetic mean since 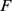 is not a rate and should match the external thermodynamic force given by Eq. (1) in stationary states. The quantity 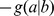 is the free energy change in units of 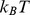 (or the negative of entropy production) for the addition of a nucleotide 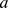 against 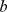, with which the backward rates 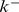 are expressed in terms of forward rates 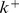 via
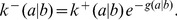
(8)


The dependence of 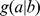 on concentrations of monomers is nontrivial because four NTPs compete for a single site. Relative stabilities of correct versus mismatched base pairs (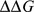), in contrast, are expected to be largely insensitive to concentrations. The following form of 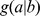 reflects this expectation [34]:
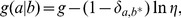
(9)where 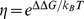, and the parameter 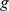 accounts for the dependence of 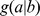 on concentrations (with mole fractions of NTPs assumed to be maintained equal during variations of [NTP]/[PPi]). With 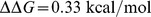, we have 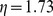 at 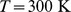. Further physical insights into the free energy parameter 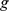 can be gained by considering the condition of equilibrium (see Methods).

It can be shown that 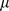 ranges from a minimum 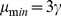 far from equilibrium (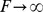, 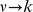), leading to Eq. (2b), to a maximum 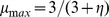 at equilibrium (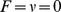) (see Methods). This variation of 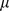 with varying thermodynamic force 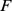 can be interpreted as follows: near equilibrium, both the correct (faster) and incorrect (slower) incorporation steps are balanced by their reverse steps, leading to comparable net incorporation statistics. Far from equilibrium, the reverse rates become negligible and the faster correct incorporation dominates.

In Figure 3A, we show that the mean field theory is exact for arbitrary symmetric template models. Figure 3B supports the site-independence of 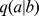 [Eq. (35) in Methods] for more general 16-parameter cases. Comparisons of the mean field theory predictions for elongation properties of pol 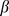 kinetics (Table 2) with simulations (Figure 3C–D) show that the theory generally gives reliable results. The Jukes-Cantor reduction of empirical rates [Eq. (4)] based on Eqs. (2) is also seen to give a good approximation over all parameter ranges, showing that the analytical theory developed in Ref. [34] provides accurate descriptions of realistic kinetics. Nevertheless, for the best numerical accuracy of predictions based on experimental kinetics, we based our main results in the following sections on stochastic simulations. Importantly, however, the mean field theory in the current application yields the definitions given by Eq. (2), in addition to the analytical limits of velocity and error rate (see Methods), which we verified exactly from simulations.

### Single-molecule properties

**Figure 4:**
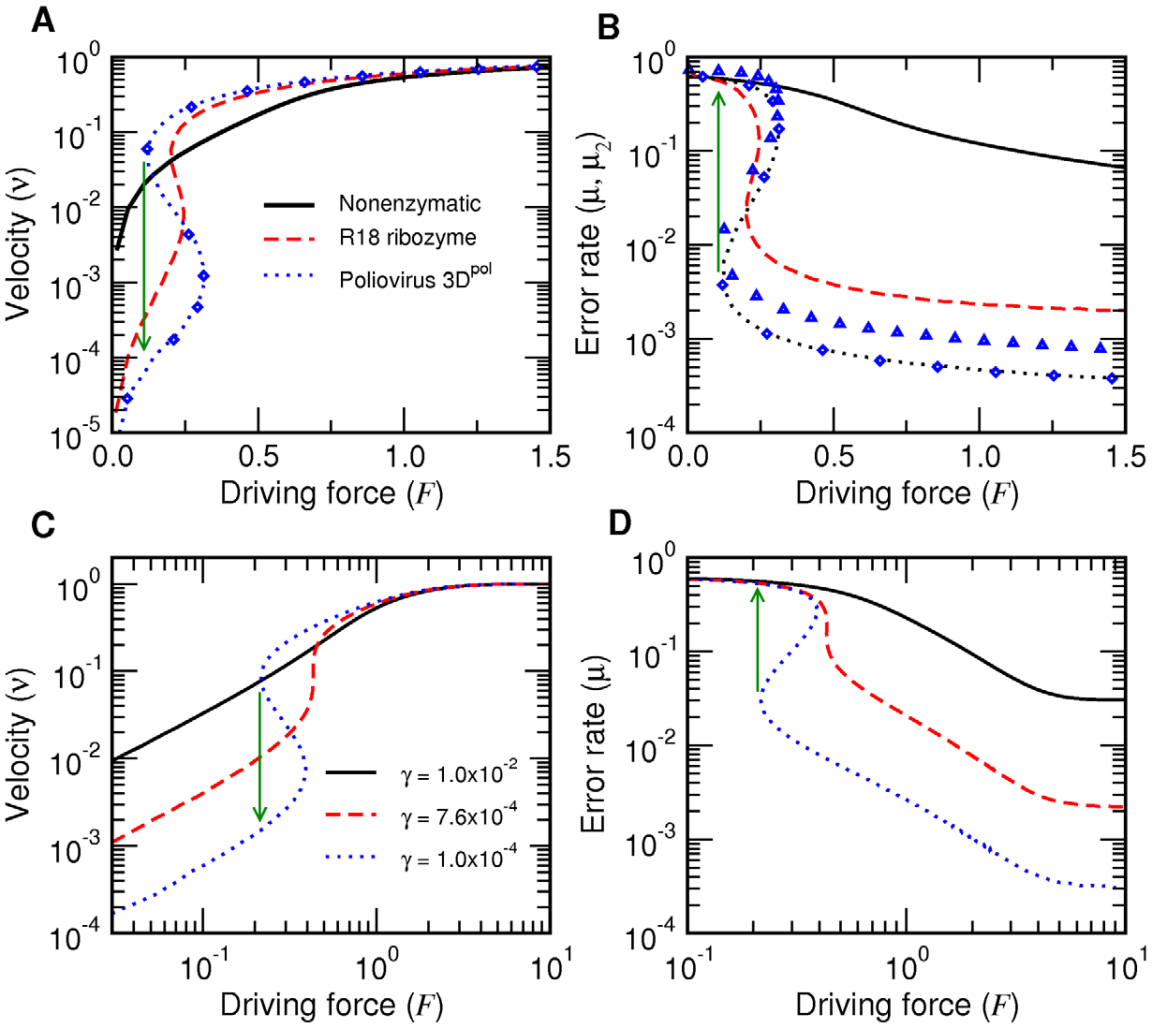
Single-molecule elongation properties as functions of 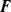. A–B: Mean RNA sequence elongation velocity 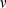 in units of 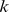 (A) and mean error rate (B) with nonenzymatic, R18 ribozyme, and poliovirus 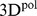 kinetics, which show supercritical, near-critical, and subcritical behaviors, respectively. Green arrows indicate discontinuous jumps for poliovirus. The diamonds denote 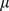 values using the poliovirus sequence (instead of random sequences for others), and the triangles indicate 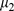 for poliovirus. C–D: Mean elongation velocity (C) and mean error rate (D) with increasing fidelity based on rescaled nonenzymatic kinetics.

We applied this single-molecule description of RNA replication to three experimental systems: nonenzymatic reactions [28], ‘Round-18’ (R18) polymerase ribozyme [9], and poliovirus polymerase (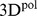) [43] (Figure 3), each representing the beginning, intermediate, and late stages of evolution. As has been previously observed in Ref. [34] for the Jukes-Cantor model, the qualitative trend shown in Figure 4 parallels that of fluids undergoing vapor-liquid transitions with decreasing temperature when pressure, volume, and temperature are replaced by thermodynamic force 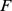, velocity 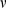, and inverse fidelity 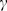, respectively. The correspondence of 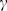 to temperature in fluids, in particular, is natural because it is a microscopic measure of randomness destroying genomic information. Figure 4A,B shows that for high inverse fidelity 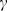 (nonenzymatic), the elongation velocity 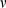 and error rate 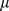 monotonically increase and decrease, respectively, with increasing 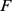. A critical point is crossed (ribozyme) as 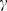 decreases, and 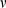 and 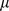 become nonmonotonic (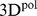) with discontinuous jumps for decreasing 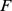 (‘evaporation’). The error rate 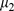 exhibits the same qualitative behavior (Figure 4B). These results verify the biological applicability of the theoretical predictions made previously in Ref. [34], based on known experimental kinetic data of systems representing key milestones of evolutionary processes (Figure 2).

The key question then is: how would these changes in the elongation behavior of RNA replication actually have occurred during the prebiotic evolution? To address this question, we modeled the increases in fidelity from the nonenzymatic starting point by uniformly rescaling the incorrect incorporation rates [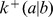, 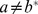] of the set of nonenzymatic kinetics (Table 1) to produce different values of 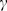. Simulations identified the critical point suggested in Figure 4A,B at 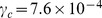 and verified the limits of error rates at and far from equilibrium predicted by the mean field theory exactly (Figure 4C,D).

### Phase behavior

**Figure 5:**
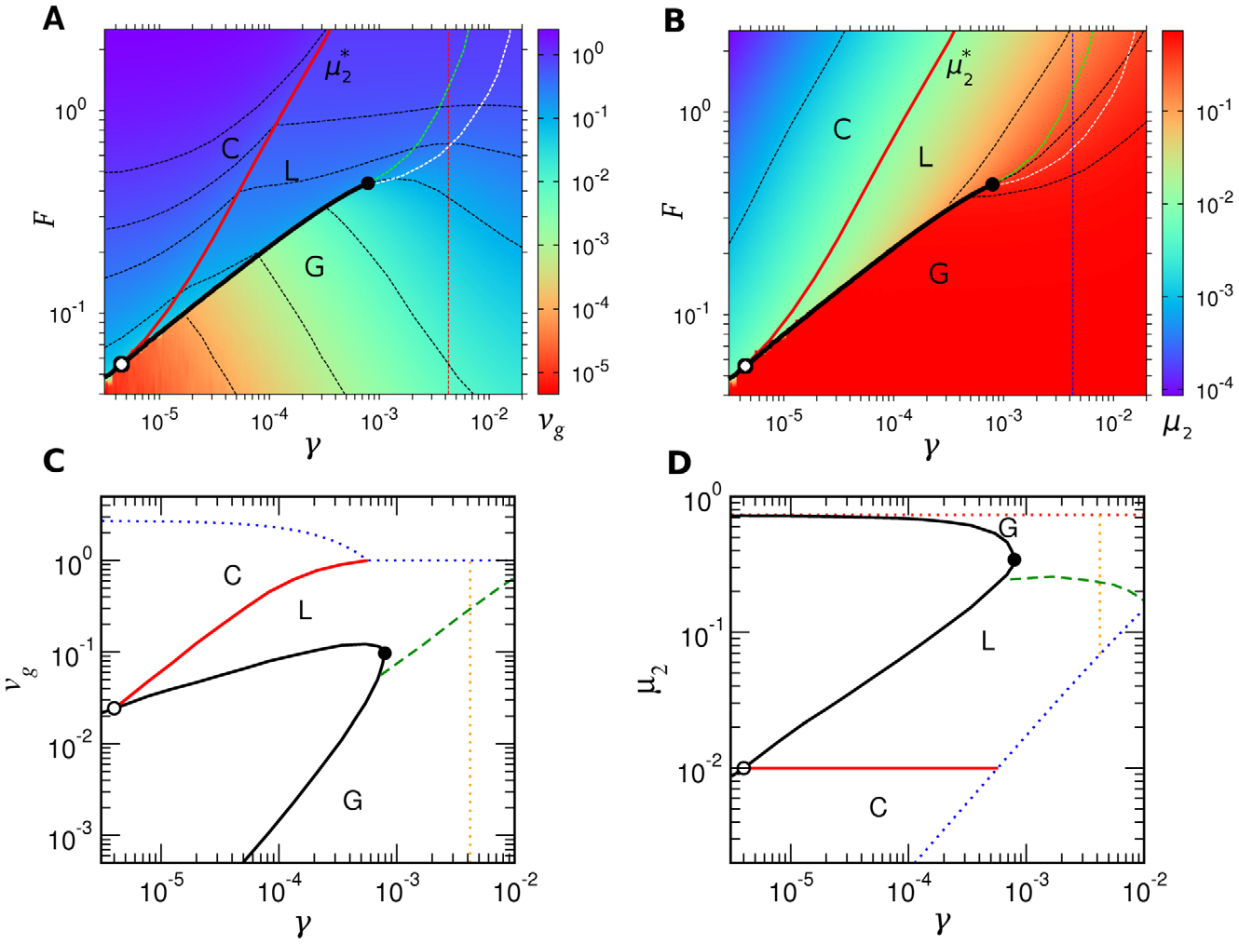
Thermodynamic phase diagrams of RNA replication. A–B: The 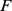-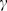 diagrams with color levels and contours (black dashed lines) representing 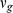 (A) and 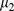 (B). The black solid lines show the spinodal terminated by the critical point (filled circles). The red solid lines show the L-C transition for 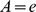 and 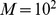, which meets the spinodal at the triple point (open circles). The white dashed lines show the boundary of 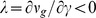 region (smaller 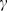 side). The green dashed lines show the analogous region of 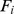 values for starvation processes (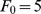). The vertical lines show the location of the nonenzymatic 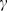 value. C–D: The 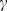-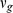 and 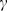-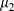 diagrams. The green dashed lines represent the 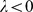 boundary. The blue dotted lines give the maximum and minimum 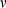 and 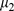, respectively, and the red dotted line in D denotes the maximum error rate at equilibrium. The fitness 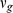 is in units of 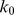.

We then scanned the variation of these phase behavior for different values of 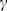 and 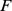 to generate the phase diagrams shown in Figure 5, which confirms that the qualitative features of the Jukes-Cantor model phase diagram [34] are preserved for empirical RNA replication. However, as opposed to the results in Ref. [34] that represent generic predictions, Figure 5 is based on empirical nonenzymatic kinetics and its uniform rescaling, with no other adjustable parameters.

The discontinuous jumps shown in Figure 4C,D correspond to the limit of stability (‘spinodal’; thick black lines) of the ‘liquid’ or L phase (high 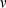-low 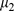 state) against the ‘gas’ or G phase (low 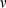-high 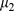 state). In equilibrium, the location of a phase transition in the phase diagram is determined by the equality of free energies of the two phases [52], [53]. Here, we adopted the assumption that if multiple stationary states exist for a given 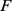, the state with higher 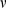 (and lower 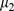) is chosen. This assumption is based on the relationship
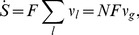
(10)connecting the entropy production rate 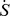 to 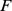 and the velocity 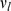 of RNA replicator 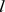 present in the system, where 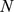 is the total number of replicators and 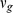 is the mean velocity (or ‘fitness’). The analogy to equilibrium phase behavior also excludes the first-order character of liquid-solid transitions, which for the current case is continuous. Equation (10) is a special case of a general relationship between nonequilibrium fluxes and conjugate forces [52]. In this formulation, a state with high 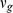 contributes more to entropy production. This assumption is consistent with the standard interpretation of the replication rate as a measure of fitness [15], [54]. The multiplicity of stationary states at a single-molecule level is supported by the recent demonstration of a real-time sequencing-by-polymerization technique [55], where it was reported that polymerases interconverted between two distinct velocities during DNA elongation for a given reaction condition (Figures 3C and S3 of Ref. [55]). A complete kinetic characterization of the 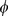-29 polymerase used in this experiment would allow us to make a more quantitative assessment of this interpretation.

### Population dynamics

In considering the thermodynamic interpretation of the population dynamics of RNA sequences, we adopt the following physical model (Figure 1): during evolutionary drifts of a random population in sequence space, a particular sequence that folds and catalyzes the replication of RNAs with the same sequence (and no others) is ‘discovered.’ (In reality, a ribozyme would more likely have had catalytic activities for arbitrary sequences. The selectivity toward its own sequence, instead, would have arisen from the need for spatial diffusion in order to act on other sequences.) This sequence therefore has a higher 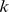 value [Eq. (2a)] compared to others, leading to the single-peak Eigen landscape [Eq. (18) below]. Our goal in this and the following subsections is to describe the growth and stability of this master sequence. In Ref. [34], the basic quasispecies theory under the single-peak landscape was combined with the theory of a single-molecule elongation. We expanded this treatment by considering different scenarios of how 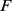 and 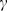 may have been distributed in RNA populations (Figure 1B).

For the inverse fidelity 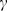, one may first assume that it is nearly uniform (or regard it as an average over replicators) in a population, as has been assumed implicitly in Ref. [34]. We also assumed that only the RNA strands with a certain polarity (analogous to the positive or negative-sense polarities of viral genomes [56]) have catalytic activities, such that a pair of replication events is necessary to reproduce a polymerase ribozyme. This feature makes the current treatment more realistic for RNA prebiotic evolution compared to those in Ref. [34]. The following derivation of the thermodynamic quasispecies theory in this subsection otherwise adopts the approach therein [34].

In a population of self-replicating RNAs with genotypes labeled by index 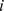, the genotype 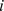 catalyzes replications with rates
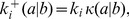
(11)where 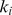 is the equivalent of Eq. (2a) for the genotype 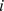 specified by a fitness landscape. The relative rate 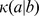 specifies the rate of addition of nucleotide 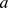 against base 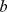, all normalized such that 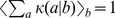. In this model, therefore, all genotypes have the same set of relative enzymatic rates for nucleotide pairs (and the same value of 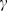), while differing in their absolute magnitude of catalysis, 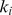. This assumption of uniform inverse fidelity is reasonable for populations with genotypes distributed within a small neighborhood of a master sequence (or a small random subspace in the absence of a master) in the sequence space. The elongation velocity 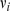 is given by
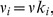
(12)where (the relative velocity) 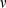 is now determined from Eqs. (5c) with 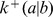 replaced by 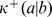, and the replication rate of genotype 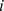 is
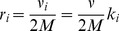
(13)because a pair of replication events requires the addition of 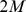 nucleotides, where 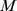 is the length of genome.

The mutation rate 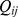 from genotype 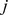 to 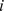 is given by
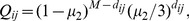
(14)where 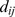 is the Hamming distance (the number of nucleotides that are different) between 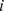 and 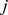. Denoting the number of individuals (of the polarity that has catalytic activity) with genotype 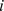 as 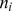, the evolving population in the Eigen model [15], [16] without constraints on the population size obeys the dynamical equation,
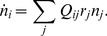
(15)At any time 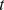, the total number of all individuals (population size) 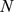 is given by 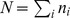, which from Eq. (15) changes via 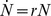, where 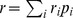 is the population growth rate (mean fitness) with the frequency of genotype 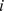, 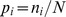. Therefore, for a given population characterized by the set 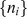, the corresponding entropy production rate is given by Eq. (10) with
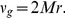
(16)


Similarly, under an idealized condition where replication occurs together with degradation [15], [17], [57], a population can evolve under a constant 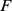 with a fixed mean population size. In this case, a replication event occurs with the same rate as the random degradation of a replicator. The evolution equation becomes
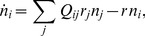
(17)such that 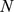 is constant. For the fitness landscape, we adopted the single-peak Eigen landscape:
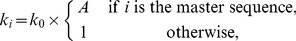
(18)where 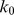 is a constant with the unit of a rate and 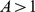 is the relative fitness of the master sequence.

Under these simplifying approximations, the standard quasispecies theory becomes applicable directly, with connections to thermodynamics made by 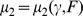 and 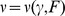. These fundamental relationships linking elongation properties to thermodynamic and kinetic parameters can be written in implicit but closed analytical forms [34] for the Jukes-Cantor model. In the numerical approach adopted here for arbitrary rates, simulations are first performed for a given set of rate constants and 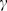 values to obtain averages of 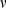, 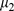, and 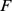 values as functions of 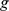, as illustrated in Figure 1 of Ref. [34]. The implicit parameter 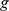 is then eliminated to obtain 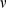 and 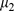 as functions of 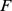 (Figure 3C–D). For the region in which multiple branches of 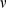 exist for a given 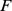, the branch with the largest 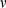 (L phase) is chosen.

In the infinite population limit, the quasispecies is either dominated by the ‘master species’ with the mean fitness 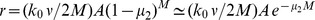, where 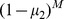 is the probability of replicating 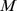 sites over two consecutive cycles without error [see Eq. (6)], or by the ‘mutant species’ with fitness 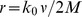. From Eqs. (13) and (16), the mean fitness is therefore given by
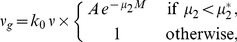
(19)where
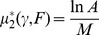
(20)denotes the threshold error rate for which 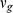 becomes the same for the master mutant species. Equation (19) implies that a constant-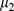 contour (red lines in Figure 5) is the ‘melting line’ separating a ‘crystalline’ (C) phase from the L phase [34]. As shown in Figure 5, the L-C transition line meets the L-G line at the triple point, below which the C and G phases meet directly (‘sublimation’). Our results show that this fairly complete analogy to the equilibrium phase behavior of fluids discovered first in Ref. [34] is indeed equally applicable in more realistic considerations of RNA prebiotic evolution.

The L-C transition line lies at the heart of the crystallization of genomes that may occur during evolutionary walks [13] in sequence space. The presence of the L phase distinct from the G phase below the critical point has an important consequence to such sequence explorations: despite the absence of a stable genome, analogous to liquid phases with short-range orders, RNAs in the L phase with 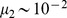 (Figure 5D) would still exhibit sequence correlations for a significantly large number of generations. We may use the Jukes-Cantor relationship between the error rate and the cumulative mean Hamming distance 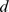 from an ancestral sequence after 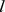 generations [35],
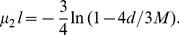
(21)A typical sequence in the G phase with 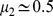 (Figure 5D), for instance, would evolve to reach 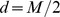 in just 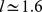 generations, on average, whereas in the L phase with 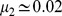, it would do so in 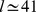 generations. Therefore, when a system ‘evaporates’ into the G phase, an ancestral sequence gets lost in a couple of generations. In contrast, conditions in the L phase, with error rates comparable to those in the C phase nearby in the phase diagram, would greatly facilitate crystallizations of viable genomes.

In interpreting the physical distinction between L and G phases, it is useful again to compare them with their analogs in equilibrium fluids, the liquid and gas phases in a container. The pressure of a fluid in equilibrium is controlled by the external force per unit area of the container, which matches the average of microscopic forces per unit area exerted by molecules on the wall interior. At high temperatures (the average kinetic energy of molecules), a given external pressure can be balanced by the mean force of a state (gas), where density is low and molecules rarely interact. The equilibrium density is then roughly proportional to pressure and inversely proportional to temperature. At low enough temperatures, a given external pressure can also be matched by a different phase (liquid) with a much higher density held together by intermolecular attractions. Both gas and liquid phases are characterized by the lack of long-range order. The sharp boundary between them appears when temperature goes below the critical value because the effect of molecular interaction renders a certain range of pressure values unstable.

Analogously, an RNA molecule replicating in a chemical reservoir is driven by the external thermodynamic force given by Eq. (1), which matches the average entropy production per monomer addition. For large 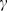 values, the replication is nearly random and the second term of Eq. (7), the sequence disorder contribution to the entropy production, is constant (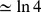), making the dependence of internal 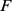 on 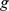 monotonic [34]. With a sufficiently small 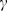, in contrast, the sequence disorder nearly vanishes, reducing the entropy production. This change is compensated by the dominance of faster correct incorporation steps, with the corresponding increase in velocity and decrease in error rates. A given value of external 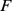 can be matched either by a state with low velocity and high errors (G phase), or by one with high velocity and low errors (L phase), each distinguished by the relative importance of the two terms in the square brackets in Eq. (7). The sharp boundary between them appears because, for intermediate values of 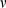, stationary states become unstable against fluctuations. The neighborhood of regimes where the C phase is stable is dominated by the L phase (Figure 5) in which the error rate is comparable to those in the C phase, if 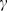 is subcritical.

**Figure 6:**
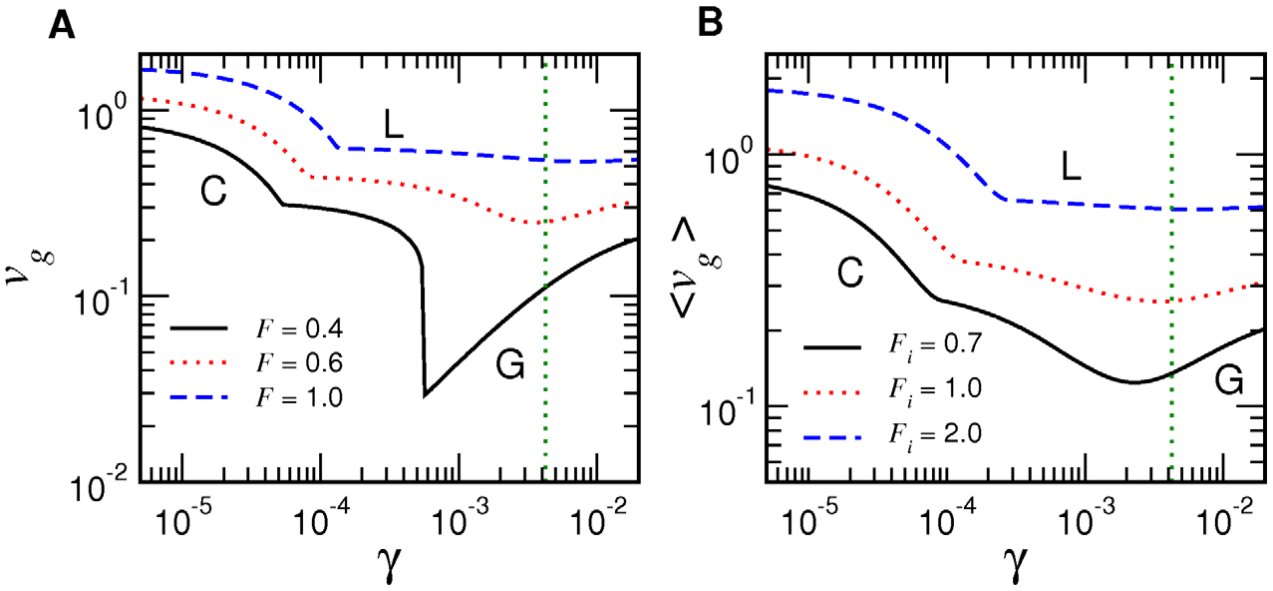
Dependence of mean fitness on fidelity. A: Mean fitness as a function of 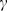 at constant 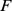. The slope 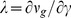 is negative below a threshold 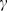 for each 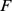 (white dashed lines in Figure 5A,B and green dashed lines in Figure 5C,D, respectively). The discontinuous jump for 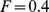 and the cusps at smaller 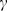 values correspond to G-L and L-C transitions, respectively. B: Mean fitness averaged over starvation processes (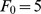) for different initial thermodynamic force 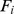 (see Figure 10). The slope 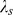 is negative below a threshold 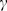 for each 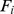 (green dotted lines in Figure 5A,B). Vertical lines represent the nonenzymatic fidelity. The fitness 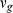 is in units of 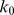.

For the population as a whole, random drifts in 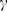 due to sequence explorations are not isotropic but, rather, are biased toward the direction of increasing 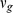. In our previous work [34], a threshold was identified within the phase diagram separating regimes where the direction of this bias shifts. We sought the analog of this threshold in Figure 5 corresponding to the evolution of RNAs, where the region in which 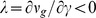 in the L phase (white dotted lines in Figure 5A, B) includes the nonenzymatic fidelity value and links it to the C phase. Inside the C phase, 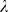 is always negative (Figure 6A). Once a population has 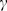 values to the left of the white dashed line in Figure 5A, random drifts in sequence space would be biased toward increasingly higher fidelity, leading to crystallization and stable genomes.

### Stochastic evolutionary dynamics

We next relaxed the assumption that 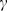 is uniform within a population (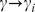 is the value for genotype 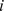). Equation (13) is then replaced by
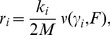
(22)for which numerical simulations have to be used. An efficient method to extract collective population dynamics of competing molecules is again provided by the Gillespie algorithm [58], which was first applied to the quasispecies dynamics by Nowak and Schuster [59]. The set of possible reactions corresponding to Eq. (17) a population can undergo are written as
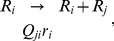
(23a)

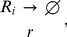
(23b)where 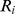 is a replicator of genotype 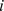. The mutation matrix is given by
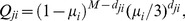
(24)where 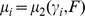 is the error rate of reactions catalyzed by the genotype 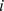.

**Figure 7:**
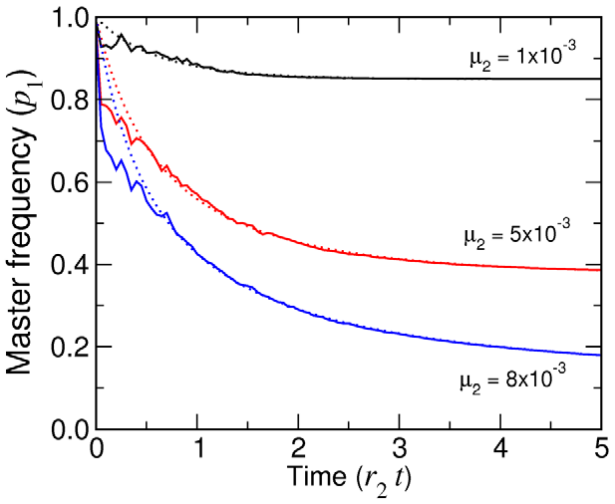
Time dependence of master sequence frequency 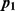
 **.** Stochastic simulation results of the Eigen model (solid lines, averaged over 1000 trajectories) are compared with Eq. (54) (dotted line), where 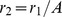 is the fitness of mutants, for 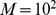 and 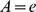. The initial condition was 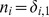.

**Figure 8:**
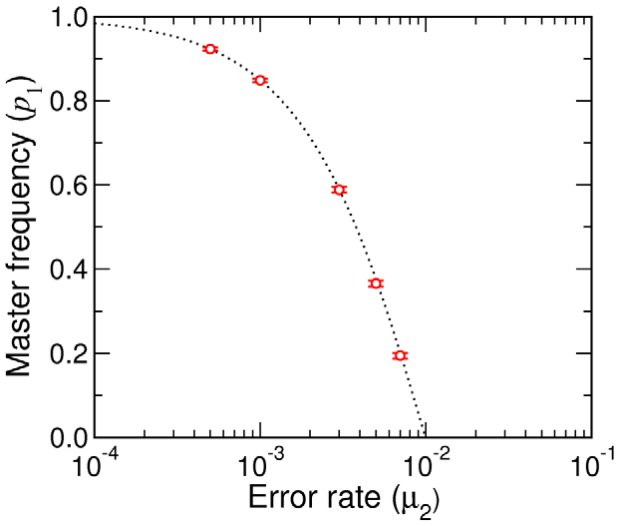
Stationary frequency of master sequence. Stochastic simulations results for the Eigen model are compared with 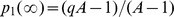. The simulations were under the condition of (approximately) constant population size (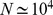) using Eqs. (23). With 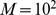 and 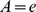, the error threshold where 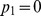 is at 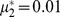. Error bars represent one standard deviations.

We tested this simulation algorithm using the special case of the exponential growth of a population with no degradation [Eq. (23a) only], uniform error rate (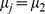), and the initial condition of single master sequence under Eq. (18) (see Methods and Figure 7). Systems with replication and degradation [Eqs. (23)] using uniform 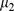 and initial population size of 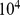 were also simulated, in which the total population size showed moderate diffusional drifts but roughly remained the same over typical trajectories, and 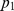 decayed to reach the steady state values (Figure 8) predicted by the infinite population result. These results show that the steady state reached in simulations depends neither on the initial conditions (single replicator or a large population) nor the boundary conditions (no degradation or constant 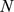).

### Crystallization kinetics

**Figure 9:**
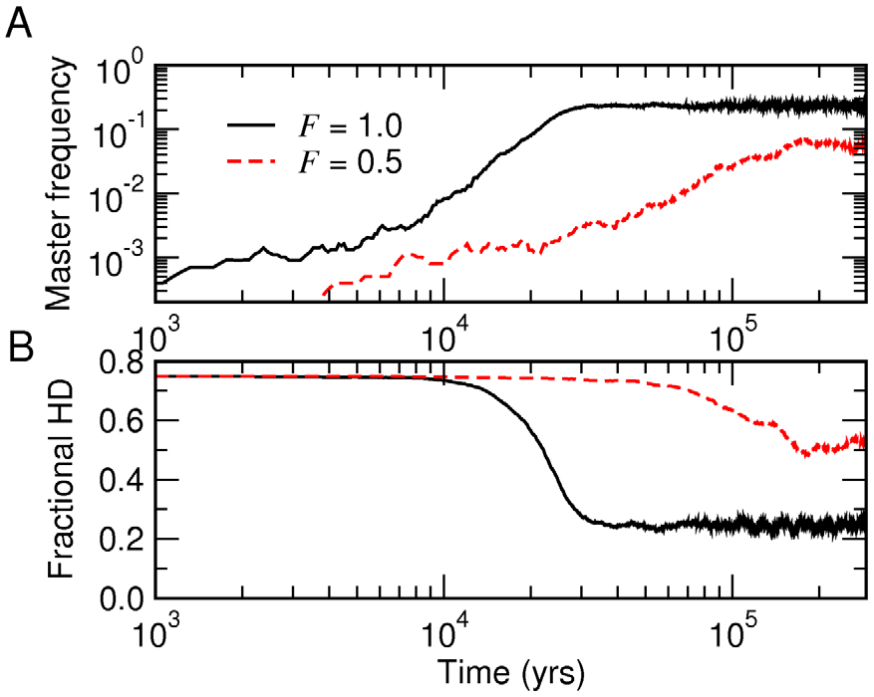
Crystallization of a genome. Stochastic simulations were used with genome length 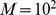 and mean base incorporation rate 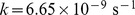. The initial population (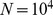) contained random sequences and a single master sequence with relative fitness 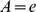. The inverse fidelity was given by Eq. (25) with 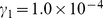 and 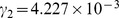. A: Relative frequency of the master sequence (initially 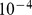). B: Average of the fractional Hamming distance (HD; 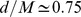 initially).

We used the constant-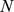 stochastic evolutionary dynamics simulations to examine the temporal evolution of quasispecies. The inverse fidelity 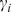 was assumed to depend on genotype 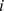 via the same form of single peak landscape as for fitness:
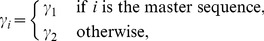
(25)where we took 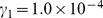 and 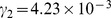 (the nonenzymatic value) in Figure 9. The time scale of simulations is set by using 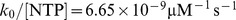 from the nonenzymatic replication (Table 1) in Eqs. (18) and (22). We assumed 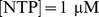 as a representative chemical environment, such that 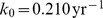.

Figure 9 shows two typical trajectories starting from an initial pool of random sequences of length 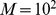, containing a single replicator designated as the master sequence. This ‘seeding’ of the population by a master sequence mimics the situation where a genotype with a significantly higher fitness is discovered during random drifts. The resulting evolution in Figure 9 is analogous to ‘crystal growths,’ in which the frequency of the master sequence steadily grows to reach a value consistent with the stability of the C phase (Figure 5): a master sequence with 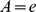 and 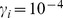 spreads and dominates the population under thermodynamic force 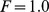. The corresponding growth under 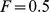, which corresponds to the vicinity of the L-C boundary in Figure 5, is much weaker and slower, suggesting that the phase diagram remains valid for inhomogeneous 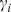. The estimated time scales in Figure 9 (based on the activated nonenzymatic rates and 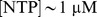) further suggest that the crystallization of a genome can occur within 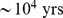 under suitable conditions. However, as in equilibrium fluids, it will never occur if thermodynamics precludes a stable C phase.

### Starvation process

We also considered an alternative setup where a population growth occurs in a closed system, which leads to an evolutionary change we refer to as the ‘starvation process.’ Similar situations were also considered in Ref. [60]. During an idealized starvation process, a single genotype is placed inside a medium containing a given amount of NTPs and PPi's with the corresponding initial thermodynamic force 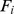. The population growth leads to the gradual depletion of NTPs and accumulation of PPi's, lowering 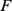. The error rate therefore increases over time. The growth of the population would come to an end when the condition finally reaches equilibrium (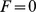). The resulting collection of RNAs in reality may then disperse into fresh media, restarting new rounds of starvation processes.

**Figure 10:**
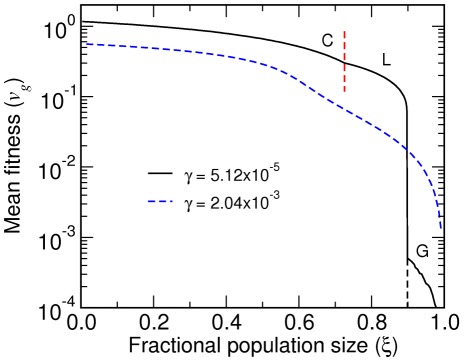
Variation of mean fitness during starvation processes. The mean fitness is shown as a function of fractional population size 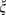. The two 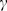 values (with 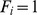 and 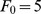) illustrate typical behavior below and above the critical point. The C-L and L-G transitions are indicated for the subcritical case.

**Figure 11:**
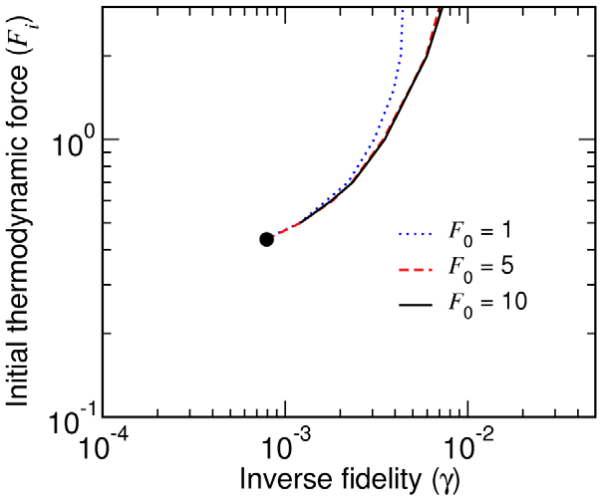
Sensitivity of fidelity threshold on equilibrium constant. The dependence on 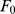 of the minimum 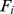 of starvation processes, for which 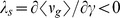, are shown.

We introduce the fractional population size 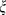 with respect to the asymptotic population reached in the limit of equilibrium (see Methods). A single process starts with 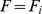, where 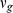 is maximum and 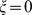, and may undergo up to two transitions (C-L and L-G) if 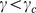 to reach equilibrium, where 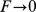, 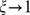, and 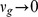 (Figure 10). In Figure 6, the mean fitness as a function of 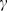 averaged over a starvation process (B) is compared with that without the averaging (A). For 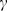 close to the nonenzymatic value (vertical dotted line), 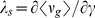 is negative in the L phase for 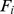 above a threshold. The dependence of the 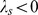 region on 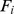 (green dashed line in Figure 5A,B) closely resembles that of the 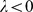 region on 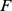 (white dashed lines in Figure 5A,B). We therefore conclude that an environment that supports repeated starvation processes with an initial 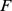 above this boundary for a given 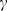 promotes evolution that lowers 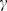. It is worthwhile to note that this conclusion was reached without invoking any significant simplifying assumptions other than the experimentally characterized kinetics of nonenzymatic replication (Table 1), thermodynamic considerations, and the quasispecies theory, except the uncertainty in values of 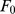. We verified that the conclusion remains valid for all possible 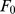 (Figure 11).

### Evolution of longer genomes

The conclusion that there was an underlying driving force biasing fidelity increases in the absence of genomes is particularly powerful because it is independent of the physical mechanisms implementing it. A likely mechanism for such changes is the evolution of error correction with the necessary increases in genome length 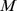. The Eigen's paradox arises because such an increase would lower 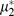 (the melting line recedes toward smaller 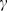 in Figure 5A,B). The melted population, however, would be driven to recrystallize a new, longer genome because 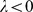 in the L phase. Growths in genome lengths most likely occurred with insertions, which is beyond the scope of our treatment that only considered base substitution errors. Saakian has studied the evolutionary model of parallel mutation-selection scheme with insertion and deletion [61]. Similar approaches combined with our findings may offer more detailed insights on how genome growths may have been facilitated by thermodynamic driving forces. In addition, we have restricted our study here to a single chemical system (RNAs). It would be of interest to apply similar approaches to more complex systems containing multiple ingredients, including peptides.

Together, our findings in Figure 5 suggest that the initial nonenzymatic fidelity of RNA lies within the threshold favoring fidelity increases. Rather than being coincidental, this feature may explain nature's choice of NTPs as the media for encoding biological information. Many possible alternative oligomers capable of templated replications have been proposed as precursors to RNAs [7]. Their corresponding monomers, however, would have had widely different fidelity values, and one system (NTPs) that happened to lie within the 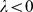 boundary presumably evolved the RNA quasispecies cloud towards smaller 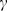, eventually crystallizing the first genomes.

## Methods

### Thermodynamic force

An elementary elongation reaction can be written as
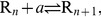
(26)where 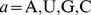 and 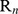 is the RNA primer of length 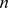. The entropy of the system plus reservoir is 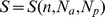, where 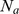 and 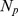 are the total numbers of monomers 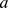 and PPi, respectively. The entropy production rate is [34], [52]

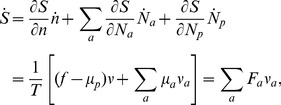
(27)where 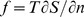 is the force acting on the growing primer (in length units of e.g., base pair rise), 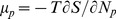, 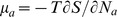, 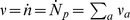, 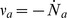 is the consumption rate of nucleotide 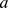. The force 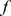 is analogous to the external tension balancing the entropic force of rubber elasticity [52]. In conditions where the external force is not controlled, it may be replaced by frictional drag on polymerases, which would depend on elongation velocity. The constant 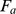 is given by
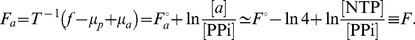
(28)In Eq. (28), 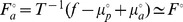, and in the third equality, we have assumed that 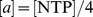. Equation (1) follows with 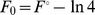, and Eq. (32) becomes 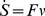.

### Equilibrium condition

A useful physical insight to 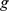 defined in Eq. (9) can be gained by considering the condition of equilibrium, which can be derived from Eq. (7) as
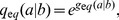
(29)or with Eq. (8), 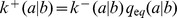, the detailed balance. In equilibrium, on the other hand, we can calculate 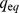 by considering a two-level system with a ground state and 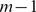-fold degenerate excited states with energy gap 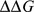 (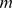 is the total number of NTP types; 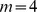 in the main text):
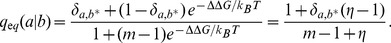
(30)Comparing this with Eqs. (9) and (29), we obtain
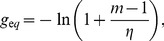
(31)which we also verify in the next subsection directly from the mean field theory. We may interpret 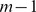 and 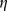 in Eq. (31) as the entropic and energetic factors for mismatches: they are costly individually (by a factor of 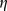) but there are many of them (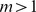). Equation (29) shows that the free energy parameter 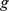 is defined with a constant term such that it absorbs the partition function that normalizes 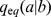 in equilibrium. The mean field theory generalizes Eq. (29) into Eqs. (5a) and (8) for nonequilibrium conditions.

### Mean field theory of RNA replication

Previously, we derived a mean field theory for the templated replication and showed with numerical tests that it was exact for the two-parameter Jukes-Cantor model rates [34]. Here, we reproduce the analytical derivation and expand it to show that the mean field theory becomes exact for symmetric template models (i.e., four-parameter models with the set of 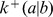 for all 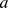 independent of the identity of 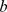), of which the Jukes-Cantor model is a special case. We also test this conclusion by comparing the analytical results with numerical simulations (Figure 3). The particular version of the mean field theory we adopted for general rates in this paper [Eq. (5)] can then be considered as a generalization of these expressions.

Considering the elongation of RNA depicted in Figure 1A, the master equation for the probability 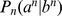 to have a chain with length 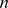 and sequence 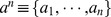 under a template 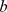 (assumed infinitely long; may refer to the whole template sequence or a single base) can be written as
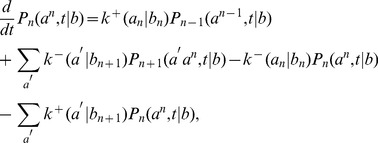
(32)where 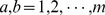 (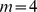 and 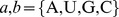 in the main text). We introduce the reduced distribution [32]

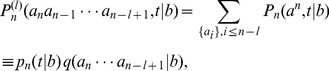
(33)where 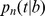 is the probability to find the chain with length 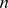 at time 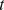 under the given template 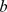, and 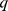 is the conditional probability of having the indicated sequence for the given chain of length 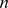. In writing 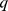 as time independent, we have implicitly assumed the stationary limit where 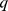 represents the asymptotic monomer distributions near the terminus of the growing chain. Our numerical simulations confirm the existence of such distributions for 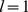. Conversely, the chain length distribution 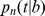 supports a peak, 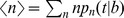, moving with a constant velocity, 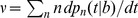, which depends on the entire template sequence 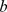 in general.

A special case of particular interest is the symmetric template models, for which the set of rates would be specified completely by at most 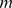 parameters instead of 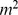. The simplest example is the two-parameter model [34] given in Eq. (4). For such models, we may write
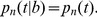
(34)The physical interpretation behind Eq. (34) is that the monomer addition and deletion at the 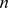
'th site on the template are solely determined by the set of rates 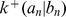, which are independent of the identity of 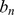. The probability for chain growth, therefore, should be independent of template sequences. We also assume that
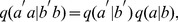
(35)which is expected because the rates 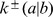 are all local in their dependence on nucleotides. Numerical tests suggest that Eq. (35) is generally valid for arbitrary rate constants (Figure 3B).

Using Eqs. (34), (35) and summing both sides of Eq. (32) over 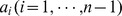, we have
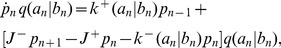
(36)where
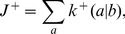
(37a)

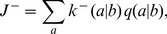
(37b)are the total fluxes for chain growth and shrinkage. Any expression involving summations over 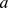 of 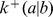 is independent of 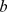 for symmetric template models.

Equation (36) is valid for any 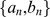, which we replace by 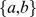. We note that 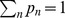, 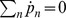, and 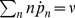. If we sum both sides of Eq. (36) over 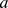 using 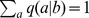, multiply by 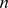, and sum over 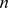, we get
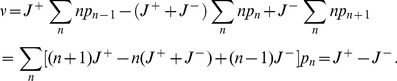
(38)If we sum both sides of Eq. (36) with respect to 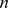,
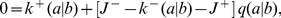
(39)or with Eq. (38),
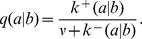
(40)


Equations (37b), (38), (40), and 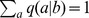 which was used in the derivation, form a set of self-consistent equations for 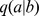 [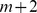 equations, 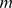 copies of Eq. (40), the normalization, and Eq. (37b); for 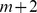 unknown, 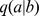, 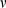, and 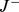]. Because 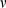 is independent of 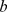, this set of equations is most conveniently solved by imposing the normalization condition to Eq. (40) to determine 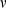:
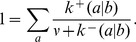
(41)We note that Eq. (41) leads to a unique 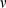 independent of 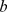 because of the symmetry of rates with respect to 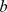. The mean error rate is given by
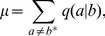
(42)again independent of 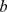. In Figure 3A, we test Eqs. (40) and (41) with an example set of symmetric template model rates. The comparison with numerical simulations shows that the expressions are exact for symmetric template models. We also tested the factorization assumption, Eq. (35), for the general case (pol 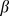 kinetics, Table 2) in Figure 3B, which suggests that it is generally valid.

Without the symmetry of kinetic rates with respect to template base identity, the main difficulty in the analytical treatment is that Eq. (34) is no longer valid, and the velocity 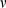 depends on the entire template sequence. There are a number of ways to generalize the exact expressions, Eqs. (40) and (41), into cases where the kinetic rates do depend on the identity of template bases. One way, demonstrated in Ref. [34], is to introduce averages over template bases to Eq. (36) to symmetrize 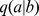 and 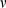 over the distribution of 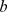. An average over 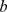 of the right hand side of Eq. (41) determines the mean velocity 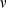 independent of 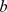 (Eq. (12) of Ref. [34]). Here, we used a different approach, generalizing Eq. (40) into Eq. (5a), which introduces a template-dependent velocity 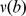. We found this mean field theory to give better agreements with simulations for asymmetric rates especially when combined with averages over 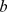 defined as the harmonic mean, i.e., Eq. (3). Because harmonic means are not additive, the summation must precede the average in Eq. (5d) within the current approach. Equation (5) becomes exact for symmetric template models.

In applying the mean field theory expressions, all forward rates are first scaled with 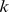 [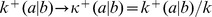], and Eq. (41) is solved for each 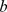 to find 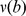. Although it is a quartic equation with respect to 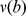, there was always only one solution for 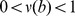. The mean velocity 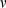, error rate 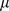, and thermodynamic force 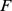 then follows from Eqs. (5c), (5d), and (7). The dependence of 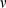 and 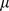 on 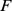 are obtained by treating 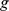 as a parameter to be eliminated (Figure 3) [62].

### Limiting behavior of elongation properties

Equilibrium is reached when 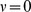 and 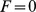, which occurs when 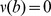 for all 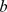: from Eqs. (5a), (8), and (9), we verify Eqs. (29) and (31), and the maximum error rate is
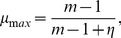
(43)which would be equal to 3/4 if 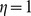 and 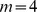. Far from equilibrium, where 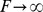, 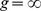 and 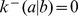, Eqs. (5a) and (41) give 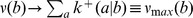 and 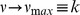, where 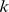 is given by Eq. (2a). The error rate in this limit approaches its lower bound,
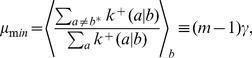
(44)which defines the inverse fidelity parameter 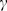 via Eq. (2b). The analogous limits of 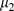 can also be calculated from Eq. (6):
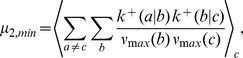
(45)and
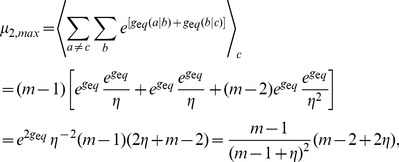
(46)where in the second equality in Eq. (46), the average over 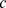 was omitted because 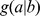 is symmetric [Eq. (9)] and the three terms in the square brackets correspond to 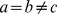, 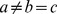, and 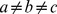 cases, respectively. For 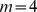, 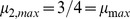 if 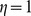, and 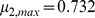 with 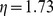 (dotted red line in Figure 5D).

### Stochastic simulation of single-molecule elongation

Numerical simulations of single-molecule RNA replication kinetics were performed with the Gillespie algorithm [58] applied to Eq. (32) [32]. A sufficiently long template sequence 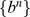 was pre-generated for the simulations using random sequences with equal distributions 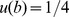. The initial condition was chosen as 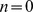 (with rate constants assigned arbitrarily for 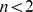), and only conditions that lead to positive velocities were considered. For a given set of 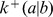 [or 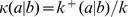], a value of 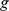 is chosen, Eq. (8) is used to generate 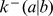, and simulations are run to obtain 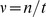, where 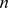 and 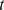 are the length of chain grown and time elapsed, respectively. The error rates 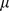 and 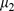, and the thermodynamic force 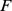 are obtained by first calculating 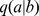 over the chain and using Eqs. (5d) and (7). This procedure is repeated for different values of 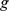 to yield 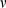 and 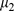 as functions of 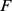. Typically, simulations were run up to 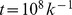 and properties were averaged over the entire chain grown. For poliovirus 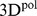, simuations were also performed using templates generated by repeating the poliovirus sequence [63] (diamonds in Figure 4B).

### Stochastic simulation of evolutionary dynamics

In the stochastic form of the Eigen model given by Eqs. (23), the total rate of transformation at a given time 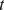 is
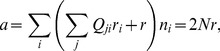
(47)where 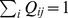 was used. In a simulation, a random number 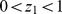 with a uniform distribution is drawn, and
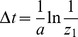
(48)determined the time 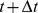 of the next replication/degradation event. A second random number 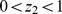 was drawn next, which chooses one (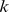, 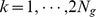) of 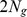 reactions (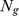 replications and 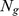 degradations, where 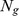 is the total number of distinct genotypes present within the population) from Eqs. (23) following Ref. [58]:
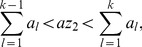
(49)where
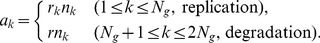
(50)For a degradation event, the number of replicators is updated as 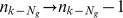. For a replication, a progeny genotype 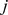 is produced from 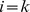 by attempting to mutate each nucleotide into 3 different bases with probability 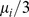, followed by the update 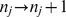. Since the total number of possible genotypes 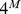 is exponentially large for even moderate values of 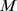, exact enumerations of 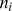 for all possible genotypes was avoided. Instead, the simulation proceeded by first creating from the initial distribution a list of genotypes for which 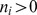, and adding newly encountered genotypes to the list as mutations occurred.

### Test case for quasispecies dynamics

For testing the Gillespie simulation of RNA population dynamics, we used the single-peak Eigen landscape (18) without back-mutation, for which the quasispecies dynamics (15) can be easily integrated. Although more advanced methods pioneered by Saakian and coworkers [17], [20]–[22], [24] allow exact analyses of the Eigen model, the following simple treatment suffices for our purpose of testing numerical simulations because for moderately large 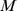, the effect of back-mutations become negligible. Writing a vector 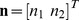, where 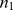 and 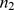 are the total numbers of individuals with the master sequence and mutants, respectively, and ignoring back-mutations, Eq. (15) can be written as
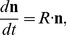
(51)where
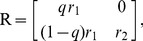
(52)and 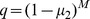. Diagonalizing 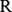 and integrating, we get
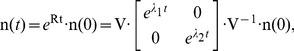
(53)where 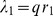 and 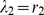 are the two eigenvalues of 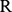 and 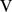 is the eigenvector matrix. We then obtain the time-dependent master sequence frequency 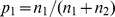,
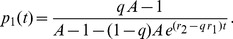
(54)
Figures 7 and 8 test numerical simulations with Eq. (54) and its stationary limit, 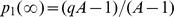, respectively.

### Starvation process

During an idealized starvation process, a single genotype is placed inside a medium containing 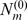 NTPs and 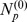 PPi's with the corresponding initial thermodynamic force 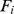. As replication progresses, 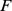 decreases via
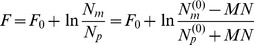
(55)assuming rapid mixing, and 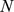 grows via 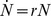. Equation (55) can be solved for 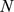 to give
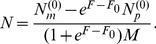
(56)Introducing the fractional population size 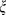 with respect to the asymptotic population 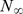 reached in the limit of equilibrium (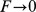),
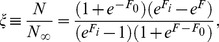
(57)or
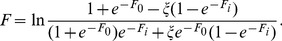
(58)Equation (58) with 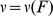 and 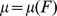 give the dependence of mean fitness on 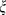 during a starvation process with the initial condition 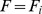. This procedure assumes that the early stages of growth with finite 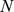 for which 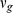 deviates from Eq. (19) make negligible contributions. The mean fitness averaged over the process is
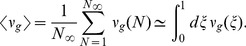
(59)This integral was performed using trapezoidal rules to obtain Figure 10.
